# Associations of Multiple Serum Tumor Markers with Radiological Response and Overall Survival in Patients with Advanced Gastric Cancer Receiving First-Line Palliative Systemic Therapy

**DOI:** 10.3390/jcm15187161

**Published:** 2026-09-15

**Authors:** Hongsik Kim, Inyoung Jang, Sun Kyung Lee, Min Kwan Jo, Jungmi Kim, Jun Su Lee, Yook Kim, Jisun Lee, Dae Hoon Kim, Hyo-Yung Yun, Hye Sook Han

**Affiliations:** 1 Department of Internal Medicine, Chungbuk National University Hospital, Chungbuk National University College of Medicine, Cheongju 28644, Republic of Korea; kimhs5186@gmail.com (H.K.);; 2 Department of Medicine, Chungbuk National University College of Medicine, Cheongju 28644, Republic of Korea; 3 Department of Obstetrics and Gynecology, Chungbuk National University Hospital, Chungbuk National University College of Medicine, Cheongju 28644, Republic of Korea; 4 Department of Radiology, Chungbuk National University Hospital, Chungbuk National University College of Medicine, Cheongju 28644, Republic of Korea; 5 Department of Surgery, Chungbuk National University Hospital, Chungbuk National University College of Medicine, Cheongju 28644, Republic of Korea

**Keywords:** gastric cancer, serum tumor markers, radiological response, overall survival

## Abstract

**Background**: Serial serum tumor-marker testing is widely used in clinical practice, but its relationship to concurrent imaging response and survival in advanced gastric cancer (AGC) remains uncertain. We evaluated five serum tumor markers—carcinoembryonic antigen (CEA), carbohydrate antigen (CA) 19-9, CA72-4, CA125, and tissue polypeptide antigen (TPA)—in patients receiving first-line palliative systemic therapy. **Methods**: We retrospectively analyzed 140 patients with unresectable locally advanced or metastatic gastric adenocarcinoma treated between 2021 and 2024. **Results**: Baseline positivity rates for the individual markers ranged from 43.6% to 73.6%, and at least one marker was elevated in 90.0% of patients. Baseline CA125 positivity was associated with a lower objective response rate compared to CA125 negativity (29.7% vs. 55.3%). Among the 117 patients with complete serial measurements of all five markers, decreases in CEA, CA72-4, CA125, and TPA were more frequent in responders than in nonresponders. Individual marker decreases showed limited discriminatory performance, whereas multi-marker thresholds showed complementary operating characteristics: a decrease in ≥2 markers had a sensitivity of 89.7%, while decreases in all five markers had a specificity of 93.2%. Patients with ≥3 elevated baseline markers had shorter median overall survival than those with <3 elevated markers (8.0 vs. 18.8 months; HR, 2.269; *p* < 0.001). **Conclusions**: Multi-marker assessment may provide complementary information at the time of radiological response assessment, but these exploratory findings require prospective and external validation before clinical use.

## 1. Introduction

Gastric cancer remains a major cause of cancer morbidity and mortality worldwide [1]. Patients with unresectable locally advanced or metastatic disease are primarily treated with systemic therapy, yet long-term outcomes remain limited despite recent therapeutic advances [2,3]. Radiological assessment remains the standard method for evaluating treatment response, but it is performed at discrete intervals and does not capture all aspects of disease biology. Biomarkers that can complement imaging and provide prognostic information could therefore be useful in the management of advanced gastric cancer (AGC).

Several serum tumor markers are routinely measured in patients with gastric cancer. Carcinoembryonic antigen (CEA), carbohydrate antigen (CA) 19-9, CA72-4, and CA125 have been associated with tumor burden, metastatic patterns, treatment response, and survival [4,5,6,7,8,9]. Tissue polypeptide antigen (TPA), which reflects cytokeratin turnover, may capture biological information that differs from that represented by conventional markers [10]. However, the sensitivity and specificity of any single marker are limited, and evidence supporting the combined evaluation of multiple markers during first-line palliative systemic therapy remains sparse.

Serum marker testing is inexpensive, minimally invasive, and readily repeatable during systemic therapy. Rather than relying on a single marker, simultaneous evaluation of several markers may better reflect the heterogeneity of AGC. Whether the number of elevated markers at baseline and concordant changes in multiple markers during treatment are associated with radiological response and survival has not been well defined.

Accordingly, the primary objective of this study was to assess the association between changes in five serum tumor markers (CEA, CA19-9, CA72-4, CA125, and TPA) from baseline to the first response assessment and concurrent radiological response to first-line palliative systemic therapy. Secondary objectives were to examine the association between baseline marker positivity and radiological response and to evaluate the prognostic association between the number of elevated baseline markers and overall survival.

## 2. Materials and Methods

### 2.1. Study Design and Patients

**Figure 1:**
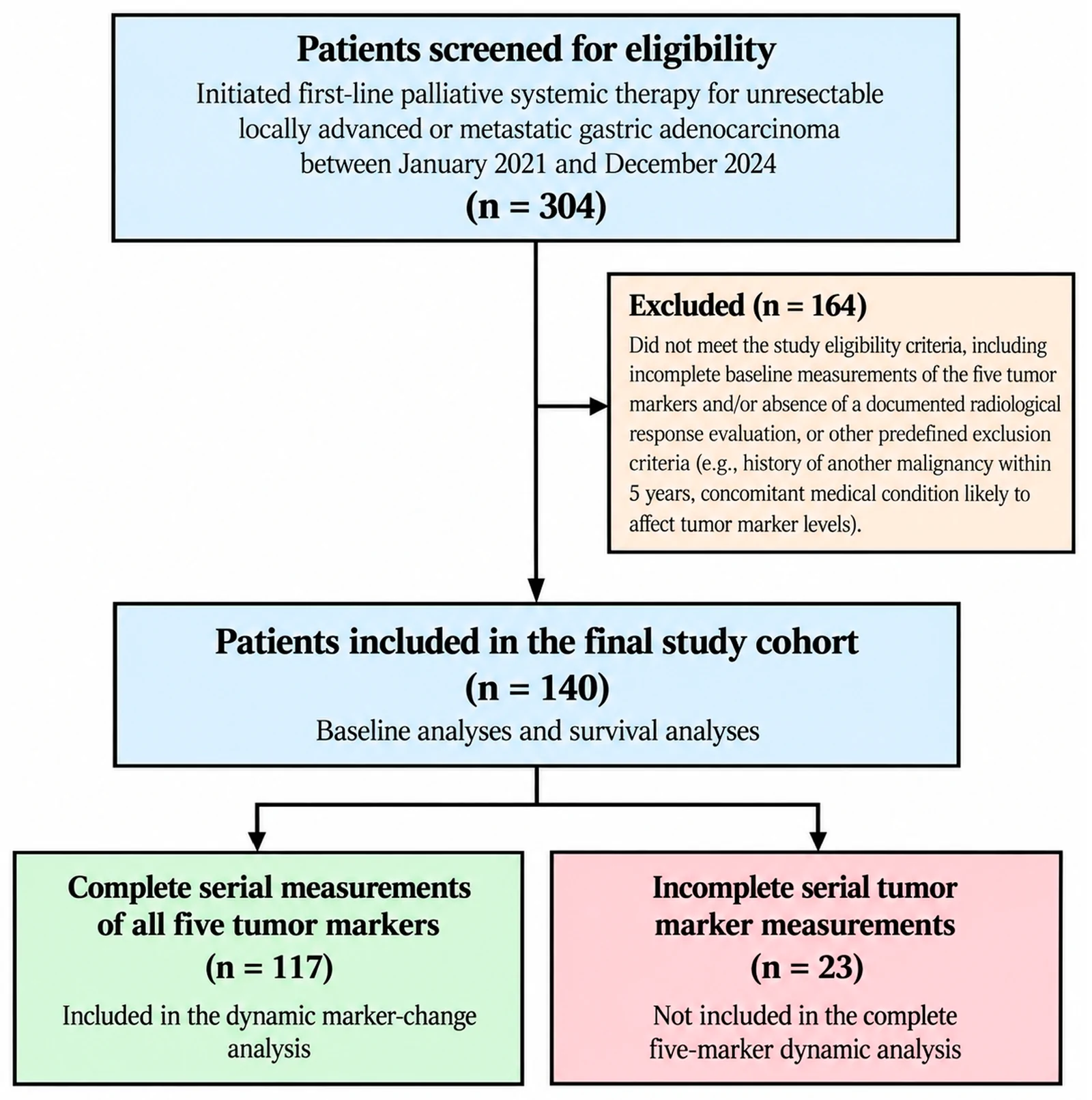
Patient flow diagram.

We retrospectively screened 304 patients with histologically confirmed unresectable locally advanced or metastatic gastric adenocarcinoma who initiated first-line palliative systemic therapy at Chungbuk National University Hospital, Korea, between January 2021 and December 2024. Eligible patients had baseline serum measurements of CEA, CA19-9, CA72-4, CA125, and TPA and at least one documented radiological response evaluation. Patients were excluded if they had a history of another malignancy within 5 years before the diagnosis of gastric cancer or a concomitant medical condition considered likely to affect serum tumor marker levels. A total of 140 patients met the study eligibility criteria and were included in the final cohort (Figure 1). Baseline clinicopathological characteristics and treatment data were obtained from medical records.

### 2.2. Treatment and Response Evaluation

All patients received first-line palliative systemic therapy for unresectable, locally advanced, or metastatic gastric adenocarcinoma. Treatment regimens were selected at the discretion of the treating physician, based on the patient’s performance status, comorbidities, biomarker status, and prevailing standards of care.

Baseline computed tomography (CT) was performed before initiation of systemic therapy, and the first follow-up CT was generally obtained 2–3 months after treatment initiation. CT scans were initially interpreted by radiologists as part of routine clinical practice before review of the corresponding serum tumor marker results. A medical oncologist involved in this study subsequently reviewed the CT images, measured target lesions, and classified the response at the first assessment according to Response Evaluation Criteria in Solid Tumors (RECIST) version 1.1 as complete response (CR), partial response (PR), stable disease (SD), or progressive disease (PD). Patients with CR or PR were classified as responders and those with SD or PD as nonresponders. Changes in serum tumor marker levels from baseline to the first response assessment were analyzed in relation to the concurrent radiological response. Because this was a retrospective study, no formal blinded independent central review was performed. The medical oncologist performing the retrospective RECIST assessment was not formally blinded to tumor-marker data, and no predefined adjudication procedure was used for discrepancies.

### 2.3. Serum Assays for Tumor Markers

Serum tumor marker levels were measured before initiation of first-line systemic therapy in all patients and again at the time of the first radiological response assessment, generally 2–3 months after treatment initiation. CEA, CA19-9, and CA125 were measured using the Alinity i CEA, Alinity i CA 19-9XR, and Alinity i CA 125 II assays, respectively, on the Abbott Alinity i analyzer (Abbott Laboratories, Abbott Park, IL, USA). CA72-4 was measured using the Elecsys CA 72-4 assay on the Cobas e 801 analyzer (Roche Diagnostics GmbH, Mannheim, Germany), and TPA was measured using the LIAISON TPA-M assay on the LIAISON XL analyzer (DiaSorin S.p.A., Saluggia, Italy). The upper reference limits were 5.0 ng/mL for CEA, 37.0 U/mL for CA19-9, 6.9 U/mL for CA72-4, 35.0 U/mL for CA125, and 75 U/L for TPA, according to the manufacturers’ reference standards. A marker was considered positive when its baseline value exceeded the corresponding upper reference limit. For combined-marker analyses, a panel was considered positive when at least one component marker exceeded its upper reference limit.

Changes in the marker levels were determined by comparing the measurements obtained during the first radiological response evaluation with the corresponding baseline values. Any reduction from baseline was classified as a decrease, whereas unchanged or increased values were classified as no decrease.

### 2.4. Study Objectives and Outcomes

The primary objective was to evaluate the association between dynamic changes in serum tumor markers from baseline to the first radiological response assessment and objective radiological response (CR/PR vs. SD/PD). Secondary objectives were to evaluate the association between baseline tumor marker positivity and objective radiological response and to assess the association between the number of elevated tumor markers at baseline and overall survival. Marker-specific percentage-change thresholds for discriminating responders from nonresponders were evaluated as exploratory outcomes using ROC curve analyses.

### 2.5. Statistical Analysis

Descriptive statistics were used to summarize baseline characteristics and tumor marker levels. Associations between baseline marker positivity and radiological response and between marker changes and radiological response were evaluated using the chi-square test. For the comparison of patients with complete and incomplete serial marker measurements, categorical variables were compared using Fisher’s exact test or the chi-square test as appropriate, and age was compared using Welch’s *t* test.

The ability of individual and multi-marker decreases to identify radiological responders was summarized using sensitivity, specificity, positive predictive value (PPV), and negative predictive value (NPV), each with 95% confidence intervals calculated by the exact binomial (Clopper–Pearson) method. Objective radiological response (CR or PR by RECIST version 1.1) was used as the reference-positive outcome. For individual markers, a decrease from baseline was considered a positive test result. For multi-marker analyses, positivity was defined as decreases in ≥2, ≥3, ≥4, or all five markers at the first radiological response assessment. As a sensitivity analysis of change magnitude, percentage change from baseline was calculated for each marker. Receiver operating characteristic (ROC) curves were used to assess discrimination between responders and nonresponders, with area under the curve (AUC) and 95% confidence intervals reported; exploratory marker-specific thresholds were selected using the Youden index. As a sensitivity analysis, marker-specific associations with radiological response were re-evaluated among patients with elevated levels of the corresponding marker at baseline, using Fisher’s exact test. Overall survival (OS) was defined from initiation of first-line systemic therapy to death from any cause or last follow-up. Survival was compared according to baseline positivity of individual markers and cumulative marker thresholds (≥2, ≥3, ≥4, and all five) using Kaplan–Meier estimates and log-rank tests. Univariate Cox proportional hazards models were used to estimate hazard ratios (HRs) and 95% CIs. The multivariable Cox model included the number of elevated tumor markers (≥3 vs. <3) as the primary variable of interest, with ECOG performance status, number of metastatic sites (≥2 vs. <2), and first-line treatment regimen included as clinically relevant covariates. The proportional hazards assumption was assessed using Schoenfeld residuals. Because ECOG performance status showed evidence of non-proportionality, an additional Cox model stratified by ECOG performance status was performed as a sensitivity analysis. All *p* values were two-sided, with *p* < 0.05 considered statistically significant. Because the marker-specific and threshold-based analyses were exploratory, no formal adjustment for multiple comparisons was applied; individual *p* values should therefore be interpreted cautiously rather than as confirmatory evidence. Statistical analyses were performed using SPSS Statistics version 25 (IBM Corp., Armonk, NY, USA).

### 2.6. Ethics Statement

The Institutional Review Board (IRB) of Chungbuk National University Hospital approved this study (IRB number: 2025-07-012) and waived the requirement for informed consent due to its retrospective design.

## 3. Results

### 3.1. Patient Characteristics and Baseline Tumor Marker Positivity

Of 304 patients screened for eligibility, 140 were included in the final cohort (Figure 1). All included patients had baseline measurements of the five serum tumor markers and a documented radiological response assessment. First-line treatment consisted of a fluoropyrimidine–platinum doublet alone in 71 patients (50.7%), a fluoropyrimidine–platinum doublet plus anti-HER2 therapy in 19 (13.6%), a fluoropyrimidine–platinum doublet plus an anti-PD-1 antibody in 41 (29.3%), and other regimens in 9 (6.4%). Baseline characteristics and serum tumor marker levels are summarized in Table 1.

**Figure 2:**
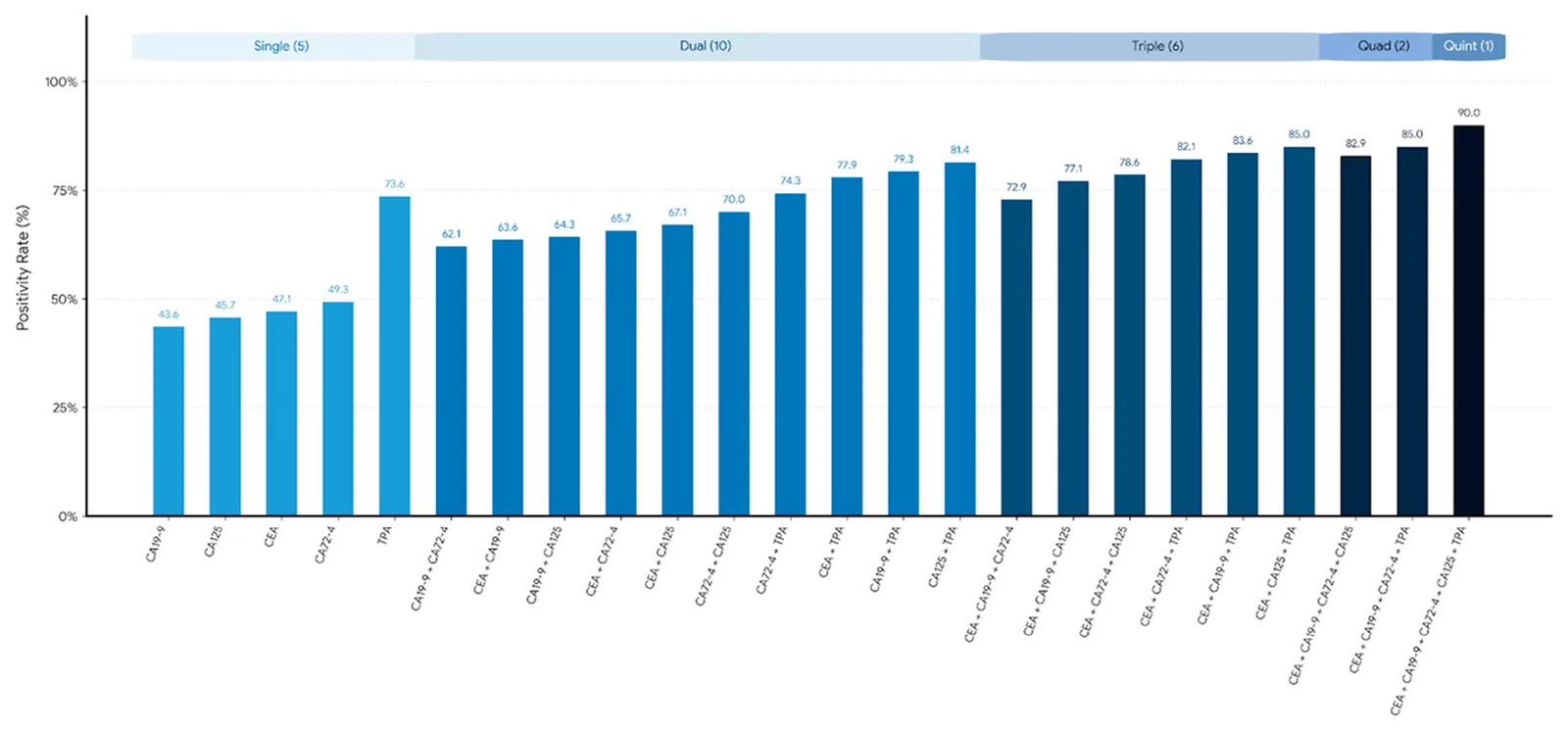
Baseline positivity rates of individual and combined serum tumor markers. Bars represent the baseline positivity rates of individual serum tumor markers and elevated multi-marker combinations. For each multi-marker combination, positivity was defined as the elevation of at least one marker above the corresponding upper reference limit. CEA, carcinoembryonic antigen; CA, carbohydrate antigen; TPA, tissue polypeptide antigen.

At baseline, positivity rates were 47.1% for CEA, 43.6% for CA19-9, 49.3% for CA72-4, 45.7% for CA125, and 73.6% for TPA. Across the evaluated combinations, the proportion of patients with at least one elevated marker ranged from 62.1% to 81.4% for two-marker panels, from 72.9% to 85.0% for three-marker panels, and from 82.9% to 85.0% for four-marker panels. At least one of the five markers was elevated in 90.0% of patients (Figure 2).

### 3.2. Association Between the Baseline Tumor Marker Positivity and the Radiological Response to First-Line Palliative Systemic Therapy

Table 2 summarizes the association between baseline marker positivity and radiological response at the first assessment. Overall, 61 of 140 patients (43.6%) achieved CR or PR and were classified as responders. Baseline positivity of CEA, CA19-9, CA72-4, and TPA was not significantly associated with response. In contrast, the response rate was lower in patients with baseline CA125 positivity than in those with CA125 negativity (29.7% vs. 55.3%; *p* = 0.002). In a multivariable logistic regression model adjusting for peritoneal metastasis, baseline CA125 positivity remained associated with a lower likelihood of objective response (adjusted OR, 0.449; 95% CI, 0.214–0.943; *p* = 0.034). Peritoneal metastasis was also associated with a lower likelihood of response (adjusted OR, 0.397; 95% CI, 0.191–0.826; *p* = 0.013).

### 3.3. Association Between the Tumor Marker Changes and the Radiological Response to First-Line Palliative Systemic Therapy

Of the 140 patients, 117 had complete serial measurements of all five tumor markers and were included in the dynamic marker analysis; 23 had incomplete serial measurements. Among these 23 patients, 11 had missing follow-up marker measurements, 10 had missing measurements in the setting of early disease progression, one was lost to follow-up, and one died from sepsis before complete follow-up marker assessment. No statistically significant differences were observed in the measured baseline characteristics between patients with complete and incomplete serial marker data. However, the incomplete-data group had a lower objective response rate (13.0% vs. 49.6%; *p* = 0.001), shorter median PFS (2.67 vs. 6.87 months; log-rank *p* < 0.001), and shorter median OS (4.17 vs. 13.2 months; log-rank *p* < 0.001) (Supplementary Table S1). Among the 117 patients with complete serial data, decreases in CEA, CA72-4, CA125, and TPA were significantly associated with radiological response, whereas the association for CA19-9 was not statistically significant (Table 3).

To examine whether decreases in markers that were within the normal range at baseline may have influenced the results, we performed an additional sensitivity analysis restricting each marker-specific analysis to patients in whom the corresponding marker was elevated at baseline. In this restricted analysis, decreases in all five markers remained associated with radiological response (Table 4).

### 3.4. Performance of Single- and Multi-Marker Changes for the Radiological Response Assessment

Using response status at the first radiological assessment as the reference standard, we evaluated the performance of decreases in individual markers and in prespecified multi-marker thresholds (Table 5). Individual marker decreases yielded sensitivities of 46.6–81.0% and specificities of 39.0–66.1%. A decrease in ≥2 markers provided the highest sensitivity (89.7%) but low specificity (33.9%), whereas decreases in all five markers provided the highest specificity (93.2%) but low sensitivity (24.1%).

In the sensitivity analysis based on percentage change, the ROC-derived decrease thresholds for discriminating responders from nonresponders were 4.8% for CEA (AUC, 0.646; 95% CI, 0.545–0.747; *p* = 0.006), 8.0% for CA19-9 (AUC, 0.643; 95% CI, 0.544–0.742; *p* = 0.008), 3.6% for CA72-4 (AUC, 0.644; 95% CI, 0.544–0.744; *p* = 0.007), 1.3% for CA125 (AUC, 0.569; 95% CI, 0.462–0.675; *p* = 0.198), and 28.9% for TPA (AUC, 0.665; 95% CI, 0.566–0.763; *p* = 0.002). Discrimination was modest for all individual markers (AUC range, 0.569–0.665), supporting interpretation of these thresholds as exploratory rather than clinically validated cutoffs.

### 3.5. Survival Outcomes According to the Baseline Tumor Marker Positivity

**Figure 3:**
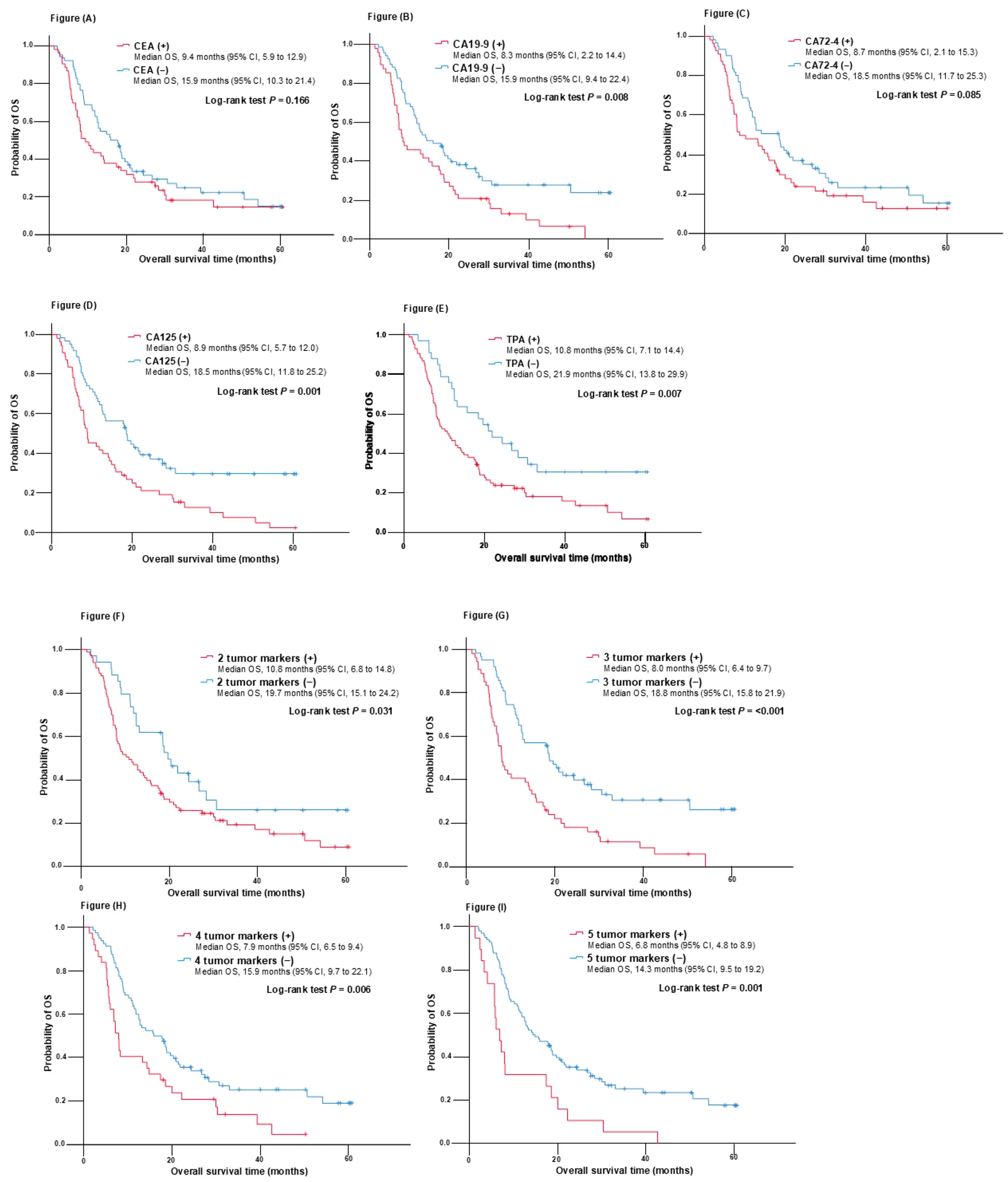
Overall survival according to the baseline positivity of individual markers and the number of elevated markers. Kaplan–Meier curves for overall survival (OS) according to baseline positivity of individual serum tumor markers: (**A**) CEA, (**B**) CA19-9, (**C**) CA72-4, (**D**) CA125, and (**E**) TPA. OS was also compared according to cumulative numbers of elevated baseline markers: (**F**) ≥2 versus <2 markers, (**G**) ≥3 versus <3 markers, (**H**) ≥4 versus <4 markers, and (**I**) all five versus <5 markers. *p* values were calculated using the log-rank test. Tick marks indicate censored observations. CEA, carcinoembryonic antigen; CA, carbohydrate antigen; TPA, tissue polypeptide antigen; OS, overall survival; CI, confidence interval.

In analyses of OS according to individual baseline markers, positivity for CA19-9 (HR, 1.748; *p* = 0.008), CA125 (HR, 1.975; *p* = 0.001), and TPA (HR, 1.952; *p* = 0.007) was associated with shorter OS. CEA (*p* = 0.166) and CA72-4 (*p* = 0.085) were not significantly associated with OS (Figure 3A–E).

A greater number of elevated baseline markers was associated with shorter OS. Median OS was 10.8 versus 19.7 months for ≥2 versus <2 elevated markers (HR, 1.686; *p* = 0.031), 8.0 versus 18.8 months for ≥3 versus <3 markers (HR, 2.269; *p* < 0.001), 7.9 versus 15.9 months for ≥4 versus <4 markers (HR, 1.824; *p* = 0.006), and 6.8 versus 14.3 months for all five versus <5 markers (HR, 2.332; *p* = 0.001) (Figure 3F–I). In univariate Cox analyses, ≥3 elevated baseline markers, ≥2 metastatic sites, ECOG PS 1, and treatment with a fluoropyrimidine–platinum doublet plus an anti-PD-1 antibody were significantly associated with OS. In the multivariable model, ≥3 elevated baseline markers remained associated with shorter OS (adjusted HR, 2.149; 95% CI, 1.421–3.250; *p* < 0.001), as did ECOG PS 1 (adjusted HR, 2.016; 95% CI, 1.317–3.086; *p* = 0.001). The association with ≥2 metastatic sites was attenuated and was no longer statistically significant (adjusted HR, 1.349; 95% CI, 0.880–2.069; *p* = 0.170). First-line treatment regimen was included in the model to account for treatment heterogeneity (Table 6). Among the 140 patients included in the Cox analysis, 114 deaths were observed. Assessment of the proportional hazards assumption showed evidence of non-proportionality for ECOG performance status (*p* = 0.008), whereas no evidence of violation was observed for the other covariates. In a sensitivity analysis using a Cox model stratified by ECOG performance status, ≥3 elevated baseline markers remained significantly associated with shorter OS (HR 2.081; 95% CI 1.373–3.153; *p* < 0.001).

## 4. Discussion

We evaluated five serum tumor markers in patients with AGC receiving first-line palliative systemic therapy and found three main patterns. First, baseline marker positivity varied substantially across markers, with TPA showing the highest positivity rate and at least one of the five markers elevated in 90% of patients. Second, decreases in CEA, CA72-4, CA125, and TPA at the first response assessment were associated with concurrent radiological response, although percentage-change ROC analyses showed only modest discrimination for individual markers. Third, the multi-marker approach showed the expected trade-off between sensitivity and specificity: requiring fewer markers to decrease increased sensitivity, whereas requiring concordant decreases in more markers increased specificity. A higher number of elevated baseline markers was also associated with shorter OS. These results support viewing the markers as complementary rather than interchangeable measures.

Previous studies of serum tumor markers in gastric cancer have focused mainly on diagnosis, surveillance after curative surgery, or a limited number of conventional markers [11,12,13,14,15,16,17]. The present study differs in that five markers were evaluated concurrently in patients receiving first-line palliative systemic therapy, with separate analyses of baseline positivity, on-treatment change, and survival. The association between ≥3 elevated baseline markers and shorter OS persisted after adjustment for ECOG performance status, metastatic burden, and first-line treatment regimen (adjusted HR, 2.149; 95% CI, 1.421–3.250; *p* < 0.001). This suggests that cumulative baseline marker elevation may carry prognostic information beyond these measured clinical factors. However, residual confounding remains possible in this retrospective cohort, and the finding should not be interpreted as evidence that marker elevation itself has an independent biological effect on survival.

The five markers may be complementary because they reflect different biological and clinicopathological features. CEA is a widely used nonspecific marker in gastrointestinal adenocarcinoma [9,18], while CA19-9 has been associated with advanced disease and metastatic patterns in gastric cancer [9,19]. CA19-9 interpretation is limited in individuals with a Lewis antigen-negative phenotype, who may have falsely low or undetectable CA19-9 levels [20]. CA72-4 is a mucin-associated glycoprotein that has shown diagnostic and prognostic value in gastric cancer [9,21]. CA125 may reflect tumor burden and mesothelial activation related to peritoneal involvement and ascites [22]. In our cohort, baseline CA125 positivity was associated with a lower response rate. This association persisted after adjustment for peritoneal metastasis (adjusted OR, 0.449; 95% CI, 0.214–0.943; *p* = 0.034), but ascites was not systematically captured and could not be included in the model. Residual confounding by ascites or other manifestations of peritoneal disease therefore remains possible, and CA125 should not be interpreted as a direct measure of treatment sensitivity. TPA consists of soluble cytokeratin fragments released during epithelial cell turnover and may reflect proliferative and cell-death activity [10]. Taken together, these differing biological associations provide a rationale for evaluating the markers jointly rather than expecting a single marker to represent the full spectrum of AGC.

Multi-marker approaches have also been explored in other gastrointestinal malignancies, where combined marker assessment has been associated with improved prognostic stratification or response monitoring compared with individual markers [23,24]. These observations provide a rationale for similar evaluation in AGC but do not establish clinical utility in this setting. In our percentage-change analyses, individual markers remained only modest discriminators of radiological response, with AUCs between 0.569 and 0.665. Accounting for the magnitude of change therefore did not identify a single marker with strong discriminatory performance. This finding is consistent with the biological heterogeneity of AGC and supports the possibility that concordant changes across several markers may be more informative than any isolated marker change. In an additional sensitivity analysis restricted to patients with elevated levels of the corresponding marker at baseline, the direction of the associations between marker decreases and radiological response was maintained. Although these findings reduce concern that the observed associations were driven solely by numerical decreases in markers within the normal range, the marker-specific subgroups were small and some response categories contained few patients. These results should therefore be considered exploratory and interpreted cautiously.

The multi-marker thresholds illustrate this trade-off. A decrease in ≥2 markers was sensitive but poorly specific for concurrent radiological response, whereas decreases in all five markers were highly specific but insensitive. The exploratory ROC-derived percentage thresholds also varied widely among markers, from approximately 1% to 29%, arguing against the use of a single universal percentage-change cutoff. PPV and NPV remained modest, and the current data do not support using marker changes as a standalone rule to replace imaging or determine imaging intervals. Because blood sampling coincided with the scheduled first radiological assessment, this study demonstrates association with concurrent response rather than prediction before imaging. Prospective studies should evaluate prespecified marker thresholds at earlier time points and determine whether marker-guided assessment adds clinically useful information beyond standard imaging.

This study has several strengths, including the concurrent evaluation of five serum tumor markers in a real-world cohort of patients with AGC treated with first-line palliative systemic therapy. Several limitations should also be considered. First, eligibility required complete baseline measurements of all five markers and at least one documented radiological response evaluation. Patients with rapid clinical deterioration or death before the first imaging assessment may therefore have been excluded, and detailed reasons for exclusion among the 164 screened patients who were not included were not systematically categorized. This may limit the generalizability of the survival analyses. Second, complete serial measurements of all five tumor markers were available in only 117 of 140 patients. The 23 patients with incomplete serial measurements had significantly lower response rates and substantially shorter PFS and OS, indicating that the missingness was unlikely to be random. Thus, the complete-case dynamic analysis may have preferentially included patients with relatively more favorable early outcomes who were able to complete the full serial tumor-marker panel at the first response assessment. This non-random missingness may have introduced selection bias and influenced the observed associations between tumor-marker decreases and concurrent radiological response. Because the missing serial measurements precluded reliable classification of marker dynamics in these patients, they could not be incorporated into the dynamic analyses without unverifiable assumptions or imputation. Accordingly, the dynamic marker findings should be considered exploratory and should not be interpreted as demonstrating clinical utility. Prospective studies with predefined serial sampling are needed to validate these associations. Third, marker changes were measured at the time of imaging and were initially categorized as any decrease from baseline. Small numerical changes may therefore reflect analytical or biological variability, particularly for values within the normal reference range. The exploratory percentage-change ROC analyses partly addressed this issue but showed only modest discrimination. In addition, radiological response assessment was not performed through a formal blinded independent review, which may have introduced assessment bias. Fourth, multiple marker-specific and threshold-based comparisons were performed without formal multiplicity adjustment; these analyses should therefore be considered exploratory. Finally, treatment heterogeneity, variation in measurement timing, and residual confounding, including unmeasured features such as ascites, may have influenced the observed associations. Independent prospective validation is required.

## 5. Conclusions

In patients with AGC receiving first-line palliative systemic therapy, concordant decreases in multiple serum tumor markers at the first response assessment were associated with concurrent radiological response, while a greater number of elevated baseline markers was associated with shorter OS. Multi-marker assessment may therefore provide complementary information at the time of response evaluation. Prospective studies with standardized sampling, prespecified thresholds, and external validation are needed before clinical implementation.

## Figures and Tables

**Table 1:** Patient characteristics (*n* = 140).

	Value (*n* = 140)
Sex	
Male	107 (76.4)
Female	33 (23.6)
Age, years	
Median (range)	66 (30–88)
≤60	42 (30)
>60	98 (70)
Pathological differentiation	
Poorly differentiated	53 (37.9)
Well-moderately differentiated	41 (29.3)
Not known	46 (32.8)
ECOG PS	
0	48 (34.3)
1	92 (65.7)
Organs with metastases	
<2	87 (62.1)
≥2	53 (37.9)
Metastatic sites	
Peritoneum	75 (53.6)
Liver	42 (30.0)
Lung	10 (7.1)
HER2 status	
Positive	20 (14.3)
Negative	120 (85.7)
First-line palliative systemic therapy regimen	
Fluoropyrimidine and platinum doublet alone	71 (50.7)
Fluoropyrimidine and platinum with anti-HER2 therapy	19 (13.6)
Fluoropyrimidine and platinum with anti-PD-1 antibody	41 (29.3)
Other regimen	9 (6.4)
Baseline serum tumor marker levels, median (range)	
CEA, ng/mL	4.3 (0.7–11,400)
CA19-9, U/mL	22.2 (2.0–120,000)
CA72-4, U/mL	6.7 (0.5–300)
CA125, U/mL	31.3 (6.6–3012)
TPA, U/mL	172.5 (20–4500)

Abbreviations: ECOG PS, Eastern Cooperative Oncology Group performance status; HER2, human epidermal growth factor receptor 2; PD-1, programmed cell death protein 1; CEA, carcinoembryonic antigen; CA, carbohydrate antigen; TPA, tissue polypeptide antigen. Patients could have metastases at more than one site; percentages are therefore not mutually exclusive.

**Table 2:** Association between the baseline serum tumor marker positivity and the radiological response.

Tumor Marker	*N*	Nonresponder, *n* (%)	Responder, *n* (%)	*p*-Value
CEA				0.549
Negative	74	40 (54.1)	34 (45.9)	
Positive	66	39 (59.1)	27 (40.9)	
CA19-9				0.375
Negative	79	42 (53.2)	37 (46.8)	
Positive	61	37 (60.7)	24 (39.3)	
CA72-4				0.75
Negative	71	41 (57.7)	30 (42.3)	
Positive	69	38 (55.1)	31 (44.9)	
CA125				0.002
Negative	76	34 (44.7)	42 (55.3)	
Positive	64	45 (70.3)	19 (29.7)	
TPA				0.963
Negative	37	21 (56.8)	16 (43.2)	
Positive	103	58 (56.3)	45 (43.7)	

Abbreviations: CEA, carcinoembryonic antigen; CA, carbohydrate antigen; TPA, tissue polypeptide antigen.

**Table 3:** Association between the change in tumor markers and the radiological response.

Tumor Marker	*N*	Nonresponder, *n* (%)	Responder, *n* (%)	*p*-Value
CEA				0.004
No decrease	62	39 (62.9)	23 (37.1)	
Decrease	55	20 (36.4)	35 (63.6)	
CA19-9				0.228
No decrease	69	38 (55.1)	31 (44.9)	
Decrease	48	21 (43.8)	27 (56.2)	
CA72-4				0.004
No decrease	52	34 (65.4)	18 (34.6)	
Decrease	65	25 (38.5)	40 (61.5)	
CA125				0.041
No decrease	43	27 (62.8)	16 (37.2)	
Decrease	74	32 (43.2)	42 (56.8)	
TPA				0.017
No decrease	34	23 (67.6)	11 (32.4)	
Decrease	83	36 (43.4)	47 (56.6)	

Abbreviations: CEA, carcinoembryonic antigen; CA, carbohydrate antigen; TPA, tissue polypeptide antigen.

**Table 4:** Association between the change in tumor markers and the radiological response among patients with elevation of the corresponding marker at baseline.

Tumor Marker	*N*	Nonresponder, *n* (%)	Responder, *n* (%)	*p*-Value
CEA				0.032
No decrease	14	11 (78.6)	3 (21.4)	
Decrease	39	17 (43.6)	22 (56.4)	
CA19-9				0.016
No decrease	16	13 (81.2)	3 (18.8)	
Decrease	32	14 (43.8)	18 (56.2)	
CA72-4				<0.001
No decrease	14	13 (92.9)	1 (7.1)	
Decrease	42	15 (35.7)	27 (64.3)	
CA125				0.041
No decrease	12	11 (91.7)	1 (8.3)	
Decrease	43	25 (58.1)	18 (41.9)	
TPA				0.007
No decrease	14	12 (85.7)	2 (14.3)	
Decrease	70	30 (42.9)	40 (57.1)	

Abbreviations: CEA, carcinoembryonic antigen; CA, carbohydrate antigen; TPA, tissue polypeptide antigen.

**Table 5:** Association between the number of decreased tumor markers and the radiological responses.

Tumor Marker	Sensitivity, % (95% CI)	Specificity, % (95% CI)	PPV, % (95% CI)	NPV, % (95% CI)
CEA	60.3 (46.6–73.0)	66.1 (52.6–77.9)	63.6 (49.6–76.2)	62.9 (49.7–74.8)
CA19-9	46.6 (33.3–60.1)	64.4 (50.9–76.4)	56.2 (41.2–70.5)	55.1 (42.6–67.1)
CA72-4	69.0 (55.5–80.5)	57.6 (44.1–70.4)	61.5 (48.6–73.3)	65.4 (50.9–78.0)
CA125	72.4 (59.1–83.3)	45.8 (32.7–59.2)	56.8 (44.7–68.2)	62.8 (46.7–77.0)
TPA	81.0 (68.6–90.1)	39.0 (26.5–52.6)	56.6 (45.3–67.5)	67.6 (49.5–82.6)
≥2 markers	89.7 (78.8–96.1)	33.9 (22.1–47.4)	57.1 (46.3–67.5)	76.9 (56.4–91.0)
≥3 markers	70.7 (57.3–81.9)	57.6 (44.1–70.4)	62.1 (49.3–73.8)	66.7 (52.1–79.2)
≥4 markers	46.6 (33.3–60.1)	76.3 (63.4–86.4)	65.9 (49.4–79.9)	59.2 (47.3–70.4)
All 5 markers	24.1 (13.9–37.2)	93.2 (83.5–98.1)	77.8 (52.4–93.6)	55.6 (45.2–65.5)

Abbreviations: CEA, carcinoembryonic antigen; CA, carbohydrate antigen; TPA, tissue polypeptide antigen; PPV, positive predictive value; NPV, negative predictive value; CI, confidence interval. The number of markers indicates the threshold number of tumor markers showing a decrease from baseline at the first radiological response assessment.

**Table 6:** Univariate and multivariable Cox proportional hazards analyses for overall survival.

Variable		No.	Univariate Analysis		Multivariable Analysis	
			HR (95% CI)	*p*-Value	HR (95% CI)	*p*-Value
Baseline elevated markers	<3 markers	71	1		1	
	≥3 markers	69	2.262 (1.555–3.290)	<0.001	2.149 (1.421–3.250)	<0.001
Number of metastatic sites	<2	87	1		1	
	≥2	53	1.556 (1.069–2.264)	0.021	1.349 (0.880–2.069)	0.170
ECOG PS	0	48	1		1	
	1	92	1.930 (1.290–2.888)	0.001	2.016 (1.317–3.086)	0.001
Peritoneal Seeding	Absent	65	1		—	—
	Present	75	1.327 (0.914–1.926)	0.137		
First-line palliative systemic therapy regimen	Fluoropyrimidine and platinum doublet alone	71	1		1	
	Fluoropyrimidine and platinum with anti-HER2 therapy	19	0.811 (0.461–1.424)	0.465	0.521 (0.285–0.953)	0.034
	Fluoropyrimidine and platinum with anti-PD-1 antibody	41	0.575 (0.366–0.903)	0.016	0.379 (0.235–0.610)	<0.001
	Other	9	0.714 (0.340–1.498)	0.373	0.462 (0.216–0.989)	0.047

Abbreviations: HR, hazard ratio; CI, confidence interval; ECOG PS, Eastern Cooperative Oncology Group performance status; HER2, human epidermal growth factor receptor 2; PD-1, programmed cell death protein 1.

## Data Availability

The data that support the findings of this study are available from the corresponding author upon reasonable request.

## Supplementary Materials

The following supporting information can be downloaded at: https://www.mdpi.com/article/10.3390/jcm15187161/s1, Table S1: Comparison of patients with complete and incomplete serial tumor-marker measurements.

## Abbreviations

The following abbreviations are used in this manuscript:

OS	Overall survival
CEA	Carcinoembryonic antigen
CA	Carbohydrate antigen
CT	Computed tomography
RECIST	Response Evaluation Criteria in Solid Tumors
CR	Complete response
PR	Partial response
SD	Stable disease
PD	Progressive disease
PPV	Positive predictive value
NPV	Negative predictive value
CIs	Confidence intervals
HRs	Hazard ratios
IRB	Institutional Review Board
