# Supplementary material for: Associations of Multiple Serum Tumor Markers with Radiological Response and Overall Survival in Patients with Advanced Gastric Cancer Receiving First-Line Palliative Systemic Therapy
Source: J Clin Med. 2026 Sep 15;15(18):7161. doi: 10.3390/jcm15187161 (PMC13607310; doi:10.3390/jcm15187161)
Supplement: Supplementary file 1 [file jcm-15-07161-s001.zip › jcm-4502171-supplementary.pdf]

Supplementary Table S1. Comparison of patients with complete and incomplete serial tumor-marker measurements

Characteristic	Complete serial measurements (n = 117)	Incomplete serial measurements (n = 23)	P-value
Age, years, mean ± SD	64.8 ± 12.1	64.3 ± 13.7	0.872
Male sex	89 (76.1)	18 (78.3)	1.000
ECOG PS 1	74 (63.2)	18 (78.3)	0.230
HER2-positive	18 (15.4)	2 (8.7)	0.528
≥2 metastatic sites	44 (37.6)	9 (39.1)	1.000
Peritoneal metastasis	62 (53.0)	13 (56.5)	0.822
Liver metastasis	37 (31.6)	5 (21.7)	0.458
Lung metastasis	8 (6.8)	2 (8.7)	0.669
≥3 elevated baseline tumor markers	54 (46.2)	15 (65.2)	0.113
First-line palliative systemic therapy regimen			0.194
Fluoropyrimidine and platinum doublet alone	55 (47.0)	16 (69.6)	
Fluoropyrimidine and platinum with anti-HER2 therapy	17 (14.5)	2 (8.7)	
Fluoropyrimidine and platinum with anti-PD-1 antibody	36 (30.8)	5 (21.7)	
Other regimen	9 (7.7)	0 (0.0)	
Objective response (CR/PR)	58 (49.6)	3 (13.0)	0.001
Median PFS, months	6.87	2.67	<0.001
Median OS, months	13.2	4.17	<0.001

Data are presented as n (%) unless otherwise indicated. P-values for categorical variables were calculated using Fisher's exact test or the chi-square test, as appropriate; age was compared using Welch's t test. PFS and OS were compared using the log-rank test. ECOG PS, Eastern Cooperative Oncology Group performance status; HER2, human epidermal growth factor receptor 2; CR, complete response; PR, partial response; PFS, progression-free survival; OS, overall survival; SD, standard deviation.
